# Factors associated with tuberculosis disease among children who are household contacts of tuberculosis cases in an urban setting in Malaysia

**DOI:** 10.1186/s12889-019-7814-x

**Published:** 2019-11-01

**Authors:** Noor Atika Azit, Aniza Ismail, Norfazilah Ahmad, Rohani Ismail, Shuhaily Ishak

**Affiliations:** 1 Department of Community Health, Universiti Kebangsaan Malaysia Medical Centre (UKMMC), Jalan Yaacob Latif, Bandar Tun Razak, Cheras, 56000 Kuala Lumpur, Malaysia; 2 Department of Health Federal Territory of Kuala Lumpur & Putrajaya, Ministry of Health, Kuala Lumpur, Malaysia

**Keywords:** Childhood tuberculosis, Active case finding, Urban setting, Active tuberculosis, Household contact

## Abstract

**Background:**

With the rise in prevalence of childhood tuberculosis (TB) globally, contact tracing should be a powerful strategy for early diagnosis and management, especially in children who are household contacts of active TB cases. Here, we aimed to determine the prevalence and factors associated with TB disease in children who are household contacts of TB cases.

**Methods:**

We used a cross-sectional study with data from the Malaysian TB Information System (TBIS) recorded from 1 January 2014 to 31 December 2017. All children aged 0–14 years who were registered in the TBIS with at least one household contact of TB cases were included in the study. Multiple logistic regression analysis was performed to calculate the adjusted odds ratio (adj. OR) and for adjusting the confounding factors.

**Results:**

A total of 2793 children were included in the study. The prevalence of active TB was 1.5% (95% confidence interval [CI]: 1.31, 1.77%). Children aged < 5 years [adj. OR 9.48 (95% CI: 3.41, 26.36) *p* < 0.001] with positive tuberculin skin test [adj. OR 395.73 (95% CI: 134.17, 1167.13), *p* < 0.001] and investigation period of > 6 weeks [adj. OR 7.48 (95% CI: 2.88, 19.43), *p* < 0.001] had significantly higher odds for TB disease.

**Conclusions:**

The prevalence of TB disease in children who were household contacts of TB cases is relatively low. However, contact tracing programmes should not only focus on children aged < 5 years and with positive tuberculin skin test results, but also be empowered to reduce the investigation period.

## Background

Until recent years, the diagnosis of tuberculosis (TB) disease in children has remained challenging. The majority of studies conducted globally have revealed that childhood TB remains under-recognised and underdiagnosed [1]. This is due to the non-specific presentation of the cases and the paucibacillary characteristics of the smear finding. The World Health Organization (WHO) classifies childhood TB as children aged 0–14 years with *Mycobacterium tuberculosis* infection [2]. The infection is acquired through the inhalation of aerosol droplets containing bacilli expectorated by an active TB patient, especially in smear-positive pulmonary TB [3]. Therefore, the likelihood of infection is higher when children are close contacts of TB cases [4].

According to the WHO, close contact is defined as a person ‘living in the same household or in frequent contact with the source case’ [5]. The source case is a TB case that results in infection among their contacts [5]. The definition of household contact commonly used in TB contact tracing programmes is ‘a person or group of people, related or unrelated to each other, who live together in the same dwelling unit and share a common source of food’ [1]. To improve the detection of TB cases, the WHO and the International Union Against TB and Lung Disease recommend that contact tracing be included in national TB programmes (NTP) [5]. This is because, in childhood TB, early detection is one of the fundamental aspects of reducing morbidity and mortality [6]. Most childhood TB cases are related to close contact with TB disease [1, 5]. A meta-analysis found that children who were household contacts had four times higher chances of acquiring infection compared to non–household contact children [7].

Currently, contact tracing is widely practised in low–TB burden countries [8]. In Malaysia, it is routinely performed after an active TB case is detected and notified to the health department. According to the Malaysian Clinical Practice Guidelines for TB, a child contact must be screened within 6 weeks of diagnosis of the index case [9]. However, previous studies conducted in Malaysia have revealed that most diagnosed TB cases remain heavily dependent on passive case detection, where patients have already developed sufficiently significant symptoms spurring them to seek medical treatment [10]. This is an undesirable outcome for any NTP, as TB is still prevalent in the country. The WHO reported that childhood TB incidence had increased to 1 million cases worldwide in 2016 [11]. Regionally, the WHO Western Pacific Region has also shown a 6% increase in total childhood TB compared to the previous report in 2015 [12].

A similar pattern has surfaced in Malaysia, with the estimated incidence increasing from 2800 cases to 3700 cases in the latest available report [11]. Even though Malaysia is classified as an intermediate–TB burden country by the WHO, TB nevertheless poses a great threat, as the neighbouring countries are high–TB burden countries, e.g. Indonesia and Thailand [11]. Moreover, Malaysia, particularly the capital city of Kuala Lumpur, receives an influx of immigrants from other high–TB burden countries from time to time. Therefore, contact tracing plays an important role in the TB prevention and control, apart from high-risk groups screening in the community. This situation indicates a further need to explore the predictors of TB disease in children who are household contacts to aid health authorities in developing a more strategic and efficient TB control and prevention programme. Hence, the present study aimed to identify the prevalence and the factors associated with TB disease in children who are household contacts of active TB cases to improve understanding of this issue.

## Methods

### Background of contact tracing in Malaysia

In Malaysia, TB is a mandatory notifiable disease under the Prevention and Control of Infectious Disease Act 1988 [9]. The district health office will receive a notification from the treating doctors within 1 week after a TB case is diagnosed. The district health officers will then conduct a case investigation and identify the contacts through interviews and a home visit. All contacts will be referred to the nearest health clinic for TB screening. To ensure that the contacts follow the referral, a written notice is issued to the contacts and to the TB team in-charge at the nearest clinic. At the clinic, the contacts will be screened by trained medical personnel, and all necessary contact information will be recorded in the Malaysian TB Information System (TBIS). This process is monitored and verified regularly by the district health officers in charge. The TBIS contains data for TB surveillance system in Malaysia. Data from the district will be analysed at the state level by the state TB organizer before compilation at the national level. The Kuala Lumpur Federal Health Office (JKWPKL) is responsible for TB disease surveillance in Kuala Lumpur, and monitors TB data from four districts: Titiwangsa, Cheras, Lembah Pantai and Kepong.

### Study setting

In 2016, the estimated population of Kuala Lumpur was 1.78 million. The city has achieved 100% urbanisation and is the most densely populated state in Malaysia. In 2016, the population density was 6891 persons per square kilometre. The estimated population aged 0–14 years was 368,600, which accounts for 20.6% of the total population in the city [13].

### Study design and population

This research adopted a cross-sectional study utilising TBIS data for the JKWPKL. All children registered in the TBIS from 1 January 2014 to 31 December 2017, aged 0–14 years and with at least one household active TB case (index case), were included in the study. The operational definition for household contact was the children and their index case sharing the same residential address. The minimum sample size was calculated based on the formula for cross-sectional studies by Fleiss (with reference to Narayanan et al. [2007]), with power of 80% and a confidence interval (CI) of 95% [14, 15]. A minimum of 240 samples were obtained.

### Study tools

The JKWPKL TBIS was used. This system contains information on all TB contacts and the index cases.

### Ethical approval

Approval for this study was obtained from the Medical Research and Ethics Committee of the Malaysian Ministry of Health (NMRR-18-642-39,752) and the National University of Malaysia Faculty of Medicine Ethics Committee (UKM PPI/111/8/JEP-2018-572).

### Outcome variables

The primary outcome was TB disease in children and was defined as children who were identified as confirmed TB cases in the TBIS.

### Independent variables

The sociodemographic characteristics included the children’s age. The children were categorised into age < 5 years and 5–14 years. Sex (male, female), race (Malay, Chinese, Indian, others) and nationality (Malaysian, non-Malaysian) were included as sociodemographic descriptors. The residential location was categorized according to the four territories of JKWPKL health offices (i.e. Kepong, Cheras, Lembah Pantai, Titiwangsa); the type of housing (low cost, non–low cost) was based on low-cost flats and houses under the people’s housing programmes (PPR). PPR houses were categorised as low-cost housing [16]. The children’s clinical characteristics were evidence of TB symptoms (yes, no), tuberculin skin test (TST) result (positive, negative, not done), sputum status (positive, negative, not done) and chest X-ray status (no lesion, cavitation, not done). The investigation period was calculated based on the date of diagnosis of the index case and the time a child was first identified as a contact and underwent investigation. This information was categorized as ≤6 weeks and > 6 weeks.

### Statistical analysis

The data were analysed using the IBM Statistical Package for the Social Sciences (SPSS) version 21. The prevalence of children who were household contacts of TB cases and who had active TB infection was calculated from the number of active cases divided by the total sample. The characteristics of the variables are described using frequency (*n*) and percentage (%) for categorical data and using the mean and standard deviation (SD) for continuous data. Simple logistic regression was used for univariate analysis. Samples with ‘not done’ TST status were excluded from this analysis. The variable(s) from the simple logistic regression analysis with *p* < 0.25 [17] was further analysed using multiple logistic regression to control for potential confounders.

## Results

Between 1 January 2014 and 31 December 2017, a total of 2793 children aged 0–14 years who were registered with the JKWPKL TBIS were identified as household contacts. The prevalence of TB disease in children who were household contacts of active TB cases was 1.5% (95% CI: 1.31, 1.77%).

The children with TB disease had a lower mean age [5.4 (SD 0.65) years] compared to children without TB disease [7.4 (SD 0.09) years]. In the children with TB disease (*n* = 43), the sex distribution was almost equal, and most were from the main ethnic group, i.e. the Malays (74.4%). Clinically, 95.3% (*n* = 41) had TB symptoms, 48.8% (*n* = 21) had positive TST, 86.0% (*n* = 37) showed lesions on chest X-ray and had negative sputum culture (90.7%). Table 1 presents the sociodemographic and clinical characteristics of the children with and without TB who were household contacts.

**Table 1:** The children characteristics who are household contact of TB cases in an urban setting in Malaysia

Characteristics	TB disease	
	Yes *n* (%)	No *n* (%)
Sociodemographic characteristics (*n* = 2793)		
Age group (years)		
< 5	22 (2.7)	806 (97.3)
5–14	21 (1.1)	1944 (98.9)
Sex		
Male	21 (1.4)	1468 (98.6)
Female	22 (1.7)	1282 (98.3)
Race		
Malay	32 (1.7)	1800 (98.3)
Chinese	3 (0.8)	367 (99.2)
Indian	2 (0.8)	258 (99.2)
Others	6 (1.8)	325 (98.2)
Nationality		
Malaysian	32 (1.3)	2442 (98.7)
Non-Malaysian	11 (3.4)	308 (96.6)
Residential location		
Kepong	17 (1.6)	1024 (98.4)
Cheras	8 (1.0)	763 (99.0)
Titiwangsa	12 (1.6)	736 (98.4)
Lembah Pantai	6 (2.6)	227 (97.4)
Type of housing		
Low cost	13 (1.4)	893 (98.6)
Non low-cost	30 (1.6)	1857 (98.4)
Clinical characteristics (*n* = 2793)		
Symptoms of TB		
No	2 (0.1)	2748 (99.9)
Yes	41 (95.3)	2 (4.7)
Tuberculin skin test		
Negative	12 (0.5)	2520 (99.5)
Positive	21 (47.7)	23 (52.3)
Not Done	10 (4.6)	207 (95.4)
Chest X-ray		
No lesion	5 (0.6)	811 (99.4)
Cavitation	37 (100.0)	0 (0.0)
Not Done	1 (0.1)	1939 (99.9)
Sputum		
Negative	39 (4.2)	888 (95.8)
Positive	3 (100.0)	0 (0.0)
Not Done	1 (0.1)	1862 (99.9)
Interval of diagnosis (weeks)		
≤ 6	23 (1.0)	2350 (99.0)
> 6	20 (4.8)	400 (95.2)

Table 2 shows the factors associated with TB disease in the children. Simple logistic regression analysis indicated six variables with *p* < 0.25, which were included in the multiple logistic regression analysis. Adjusted analysis showed that children aged < 5 years, with positive TST and investigation period of > 6 weeks had higher odds for TB disease compared to children aged 5–14-years, with negative TST and investigation period of ≤6 weeks, respectively.

**Table 2:** Factors associated with TB disease among children who are household contact of TB cases

Factors (*n* = 2576)	Crude OR^a^	95% CI	*p*-value	Adj. OR^b^	95% CI	*p*-value
Age group (years)			< 0.001			< 0.001
5–14	1			1		
< 5	3.84	(1.90,7.77)		9.48	(3.41,26.36)	
Sex			0.238	NS		NS
Male	1					
Female	1.52	(0.76,3.04)				
Race			0.381	NS		NS
Non-Malay	1					
Malay	1.41	(0.65,3.05)				
Nationality			0.003	NS		NS
Malaysian	1					
Non-Malaysian	3.20	(1.47,6.95)				
Residential location			0.331	NS		NS
Kepong	1					
Cheras	0.43	(0.16,1.19)				
Titiwangsa	0.84	(0.36,1.92)				
Lembah Pantai	1.32	(0.43,4.02)				
Type of housing			0.183	NS		NS
Low cost	1					
Non low-cost	1.77	(0.76,4.09)				
Tuberculin skin test (TST)			< 0.001			< 0.001
Negative	1			1		
Positive	191.74	(84.49,435.13)		395.73	(134.17,1167.13)	
Investigation period (weeks)			< 0.001			< 0.001
≤ 6	1			1		
> 6	4.01	(1.99,8.06)		7.48	(2.88,19.43)	

^a^Crude odd ratio using simple logistic regression; ^b^Adjusted odd ratio (multiple logistic regression using Backward likelihood method, Hosmer and Lemenshow test *p-*value 0.678*,* Nagelkerke *R*^*2*^ = 51.5%); 1 = reference, *NS* Not significant

## Discussion

The main objective of the present study was to identify the prevalence and factors associated with TB disease in children who are household contacts of active TB cases. We found lower prevalence of TB disease in the studied population compared to previous studies conducted in Southeast Asia, which reported 3.3–5.5% prevalence [18]. However, we postulate that the low prevalence in the present study represents the prevalence of TB in children in Kuala Lumpur, as it is lower than national prevalence of childhood TB of 3.1% [19]. Nevertheless, given the previous evidence of under-reporting [1, 20], the actual prevalence may be higher or the same as that in other studies in neighbouring countries. In fact, Fox et al. (2013) concluded in their meta-analysis that the overall prevalence of active TB among all household contacts is 3.1% [21]. The possibility of underdiagnosis in the child contact should be explored and may be due to failure to identify children with household contacts, and failure to screen the contact after the identification [1].

In addition, we show that children aged < 5 years had higher chances of contracting active TB. This result is consistent with that of other studies carried out globally [7, 22]. This can be explained by age and immunological response being important drivers for the progression of the disease [23]. The risk of TB infection from household contacts is high during the first 2 years of life, and the risk of dissemination in this group is the highest compared to other non-immunocompromised groups [23]. This is because infants and young children tend to have inadequate or incomplete immune responses due to age-related deficiency, with or without downregulation of the key immune responses [24]. Such a condition therefore leads to a more disseminated disease in the group [24].

Another significant finding was the TST result. In Malaysia, induration of > 5 mm and > 10 mm in immunocompromised and non-immunocompromised children, respectively, is considered a positive TST [25]. This supports the usefulness of the TST as a screening method despite its limitations [26, 27]. As microscopic diagnosis is difficult and often negative in children, TST results may provide a guide for predicting the likelihood of infection, as TB symptoms are sometimes non-specific in this age group [28]. However, the TST should not be used as the only tool for deciding on infection to increase the effectiveness of the screening programme [29].

Apart from the above, the results also highlight the importance of early contact screening once an index case has been diagnosed. According to Malaysian guidelines, the time frame of not more than 6 weeks is recommended and the contact should be followed-up in a sequential interval for 12 months after the first investigation [9]. This is because previous studies have reported that the median incubation period is 6 weeks [28, 30]. Therefore, it emphasises the need for following-up with the contact after the first investigation and highlights the importance of not delaying the first investigation, so that anti-TB prophylaxis can be administered to the high-risk groups.

In the present study, housing location and type were not significantly associated with TB in the studied population. A study on urbanisation in Malaysia suggested that high-rise flats with poor ventilation could increase the risk of TB infection [31]. Nevertheless, the present study shows that the low-cost flats provided by the government to the urban poor in Kuala Lumpur are not significantly associated with TB in the children studied. However, we did not explore the crowdedness of households, as this is not captured in the TBIS.

The limitation of this study is that it used secondary unprocessed data, where the data are collected by different officers in charge of different districts. All data were assumed to be entered in accordance with standard operating procedures outlined by the Ministry of Health and verified by the district health officers in charge.

In addition, as data were limited to the first investigation only, the exact timing of active infection could not be determined. The TBIS database should include data on subsequent follow-up for further evaluation. As the number of household contacts who contracted active TB was low, we only included the variable with event per variable of > 10 in multivariate analysis to avoid sparse data bias. Further research is suggested to follow the cohort for follow-up to evaluate the outcome of contact tracing.

## Conclusions

The prevalence of TB disease in children who are household contacts of TB cases in Kuala Lumpur is lower than the national prevalence. Children with household contacts of TB cases aged < 5 years, positive TST and index–contact investigation period of > 6 weeks were associated with TB disease. Hence, the contact tracing programme should be empowered to reduce missed opportunities for diagnosing childhood TB. Our study highlights the high-risk groups for TB prevention, the usefulness of the TST for screening, and the importance of follow-up in contact tracing for children who are household contacts of active TB cases.

## Data Availability

The data that support the findings of this study are available from Ministry of Health Malaysia, but restrictions apply to the availability of these data, which were used under license for the current study, and so are not publicly available. Data are however available from the authors upon reasonable request and with permission of Ministry of Health Malaysia.

## Abbreviations

JKWPKL: Kuala Lumpur Federal Health Office
MOH: Ministry of Health, Malaysia
TB: Tuberculosis
TBIS: Tuberculosis Information System
TST: Tuberculin Skin Test
WHO: World Health Organization
